# Radiofrequency fistulotomy: a better alternative for treating low anal fistula

**DOI:** 10.1590/S1516-31802004000400008

**Published:** 2004-07-01

**Authors:** Pravin Jaiprakash Gupta

**Keywords:** Rectal fistula, Recurrence, Anus, Radio waves, Colorectal surgery, Fístula retal, Recidiva, Cirurgia retal, Ânus, Ondas de rádio

## Abstract

**CONTEXT:**

Wide varieties of approaches are employed in dealing with low anal fistula. However, the simple method of laying open the fistula tract (fistulotomy) is still considered to be the favored one.

**MATERIALS AND METHODS:**

A modified approach to the procedure of fistulotomy is discussed. This study describes the procedure, which used a technique of radiofrequency surgery, and its outcome in 232 patients with low anal fistula. The patients were followed for a period of 15 months.

**RESULTS:**

The patients were discharged on the same day as the procedure. The mean period off work was four days. The average healing time recorded was 67 days. Four wound complications in the form of premature closure of the external wound were noted, which required trimming of the edges. Two of these wounds remained unhealed. The recurrence rate was 1.7%.

**CONCLUSION:**

In this era when the emphasis is on criteria like the minimization of hospital stay, reduction of postoperative pain, early resumption of work and low and comparable recurrence rates, there is a future for the procedure of radiofrequency fistulotomy.

## RESUMO

**CONTEXTO:**

Uma grande variedade de métodos pode ser utilizada no manejo da fístula anal baixa. No entanto, o simples método de abertura e exposição da fístula (fistulotomia) é ainda considerado o melhor deles.

**MATERIAIS E MÉTODOS:**

Um método modificado para a abordagem de fistulotomia é discutido. Este estudo descreve o procedimento, em que foi usada uma técnica de cirurgia por radiofreqüência, e o seu resultado em 232 pacientes com fístula anal baixa. Os pacientes foram acompanhados por um período de 15 meses.

**RESULTADOS:**

Os pacientes tiveram alta no mesmo dia do procedimento. A média de tempo sem trabalhar foi de quatro dias. A média de tempo para cicatrização foi de 67 dias. Quatro complicações da ferida, caracterizadas por fechamento prematuro da área externa da ferida, foram observadas, exigindo incisões da região da periferia. Duas destas feridas permaneceram abertas. O índice de recorrência foi de 1,7%.

**CONCLUSÃO:**

Nesta época em que a ênfase é dada a critérios como mínima permanência no hospital, redução da dor pós-operatória, retorno precoce ao trabalho e índices de recorrência baixos e comparáveis, existe um futuro para o procedimento de fistulotomia por radiofreqüência.

## INTRODUCTION

The classic lay-open technique is still the most favored procedure for anal fistula. Slitting the complete tract, starting from the external opening and proceeding to the internal opening, is the basis for traditional fistulotomy.^1^

However, this conventional procedure at times encounters brisk bleeding from the cut surfaces. The dissection causes unpredictable tissue trauma, thereby aggravating the postoperative pain and edema, delaying the return to work and necessitating intake of stronger doses of analgesics for longer periods. The risk of wound complications like infection, delayed wound healing and recurrence are but a few of the other areas of concern accompanying the traditional method.

Instead of using the scalpel, we have employed radiofrequency waves to perform the same lay-open procedure for treatment of low anal fistula (low trans-sphincteric and intersphincteric fistula). The surgical principle in radiofrequency surgery is that the offending tissue obstructing the path of the high-powered radio waves is destroyed. The intracellular tissue water that provides the resistance in the process is instantly vaporized without causing heat and damage, unlike what takes place in electrosurgery. This tissue vaporization also results in significant hemostasis without actually burning the tissue.^2^ In addition, there is no danger of the patient suffering any shock or burn in the process. The radiofrequency-generating unit is provided with a terminal to which different electrodes can be attached to suit the exact requirement of the procedure. We have used a ball electrode for coagulation, a needle electrode to incise the fistula tract and a round loop electrode to shave the tract tissue in our procedures. The surgical radiofrequency generator that we have been using is the dual frequency 4 MHz unit from Ellman International (Hewlett, New York).

## METHODS

The study included 232 consecutive patients with low anal fistula. A low anal fistula was taken to be one with a tract that does not extend above the level of the anal crypts and usually opens at this level into the anal canal. All of these patients were followed over a period averaging 15 months (range: 14-18 months). The following types of fistula were excluded from the study: high transsphincteric fistulas with or without high blind tract, supra-sphincteric, extra-sphincteric and horseshoe fistulas, as well as fistulas associated with inflammatory bowel disease.

### Radiofrequency fistulotomy procedure

All the patients were operated on under short-duration general anesthesia. The step-by-step approach to our procedure was as follows:

1. Injection of methylene blue dye with hydrogen peroxide^3^ into the external opening of the fistula. This helped in obtaining a clear display of the main fistula tract. It also opened up the secondary or side tracts if present.
2. While viewing through the anoscope, a directional probe was gently passed through the external opening until it came out from the internal opening, which was demarcated by the blue indentation of the dye.
3. The skin overlying the probe in the fistula tract was coagulated by moving the ball electrode over its complete length. This was found to reduce the amount of bleeding when the tract was slit opened thereafter.
4. The tract was cut opened along the line of the probe with the help of the needle electrode, switched to the ‘cut and coagulation’ mode. This reduced the bleeding while cutting and made the dissection smooth enough to avoid any drag on the tissues.
5. The wound edges were coagulated with the ball electrode kept in the ‘coagulation’ mode. This obviated the possibility of any ooze from the wound surface.
6. The tract, along with the surrounding infected fibrotic tissue was curetted with the loop electrode in the ‘cut and coagulation’ mode. As cutting and coagulation were achieved simultaneously, the brisk bleeding often encountered in the conventional knife and scissors dissection was avoided.
7. Finally, the edges of the wound were shaved, using the loop electrode to create a pear-shaped wound tapering towards the anus. The patients were discharged on the evening of the procedure. They were asked to take a tablet containing 50 mg of diclofenac sodium and 10 mg of serratiopeptidase twice a day for seven days and, thereafter, as and when they felt pain. The postoperative care consisted of the application of antiseptic cream over the wound twice a day after a warm sitz bath.

## RESULTS

The mean procedure time was 13 minutes. This was recorded by an independent observer, and was calculated as the total time required from inserting the probe in the fistula tract to the application of dressing over the wound.

The patients were discharged as soon as they became fully mobilized without the need for assistance from a nurse for personal hygiene and dressing. As the procedure was carried out under short-duration general anesthesia, most of the patients were able to go home within eight hours of the procedure.

The period off work is defined as the total time taken to return to the usual activity of domestic and social life at the patient's discretion. Patients operated on via radiofrequency fistulotomy procedures were able to resume their routine within a week of the procedure (a mean of 4 days).

A mean of 18 doses of analgesic were required, where one dose equaled one tablet of the combination of diclofenac sodium 50 mg and serratiopeptidase 10 mg.

The wound healing time was taken as the period needed for complete epithelization of the fistulotomy wound. The average time taken for wounds to heal was 67 days, ranging from 42 to 75 days.

Four patients from our study were found to have premature closure of the proximal wound while the distal end remained un-healed. The healed edges of the proximal part of the wound were slit opened under local anesthesia. Following this, the wounds healed in two of these patients. However, in the remaining two, they failed to heal. These two cases were termed ‘failure of wound healing’ rather than recurrence (0.8%).

None of the patients had any interference with continence. With a follow-up of at least 15 months, the recurrence rate was as low as 1.7% (4 out of 232 patients).

### Complications

No major complications were encountered with the radiofrequency device. However, failure to monitor the exact power requirement from the unit, in a few of the initial cases, resulted in the use of higher power rates than necessary, thereby causing smoke and charring. This was rectified in the subsequent procedures.

## DISCUSSION

Radiofrequency surgery uses a very high frequency radio wave.^4^ The tissue damage caused by radiofrequency is superficial and is comparable with the results from the harmonic scalpel and laser.

Radiofrequency surgery creates minimal collateral heat damage in the tissue, thus resulting in rapid healing and leaving no ugly scar.^5^ The unit does not require any recurring maintenance except for the normal care during its handling and use.

In comparison with other treatment techniques for anal fistula, our study produced results that were equal to or even better than obtained using these others, such as marsupialization,^6,7^ sphincter preserving procedures,^8^ coring-out technique,^9^ instillation of fibrin glue,^10-12^ flap procedures,^13,14^ excision of fistula and closure of internal opening,^15^ incision, and lay-open^16^ using rubber seton^17^ or medicated seton.^18^ The improvement came in terms of wound healing, wound complications, interference with continence and recurrence (Table 1).

**Table 1:** Comparative results from radiofrequency fistulotomy and other treatment techniques for anal fistula

Observations	Our series	Other series
Time for wound healing	67 days	Marsupialization: 42 days^6^
		Lay-open: 70 days^6^
Failure of wound healing	0.8%	Fibrin glue: 29 %7 – 100%^8^
		Flap: 36%^9^
Recurrence	1.7%	Sphincter preserving procedure: 5%^10^
		Marsupialization: 10%^11^
		Seton: 22%^12^
		Flap: 33%^13^
		Fibrin glue: 40%^14^
Incontinence for flatus	None	Lay-open: 10%^16^
		Marsupialization: 20%^6^
Incontinence for liquids	None	Excision of fistula with closure of internal opening: 11%^15^
Delayed healing	None	Open coring-out (function-preserving) technique: 15%^17^
Abscess formation	None	Medicated seton: 3-5%^18^

## CONCLUSION

The fistulotomy procedure using a radio-frequency technique has distinct advantages like a short operation time, minimal blood loss, early return to normal activity and a low recurrence rate. We found from our study that this method can serve as a useful addition for improving on the results from the conventional procedure for treating low anal fistula.
